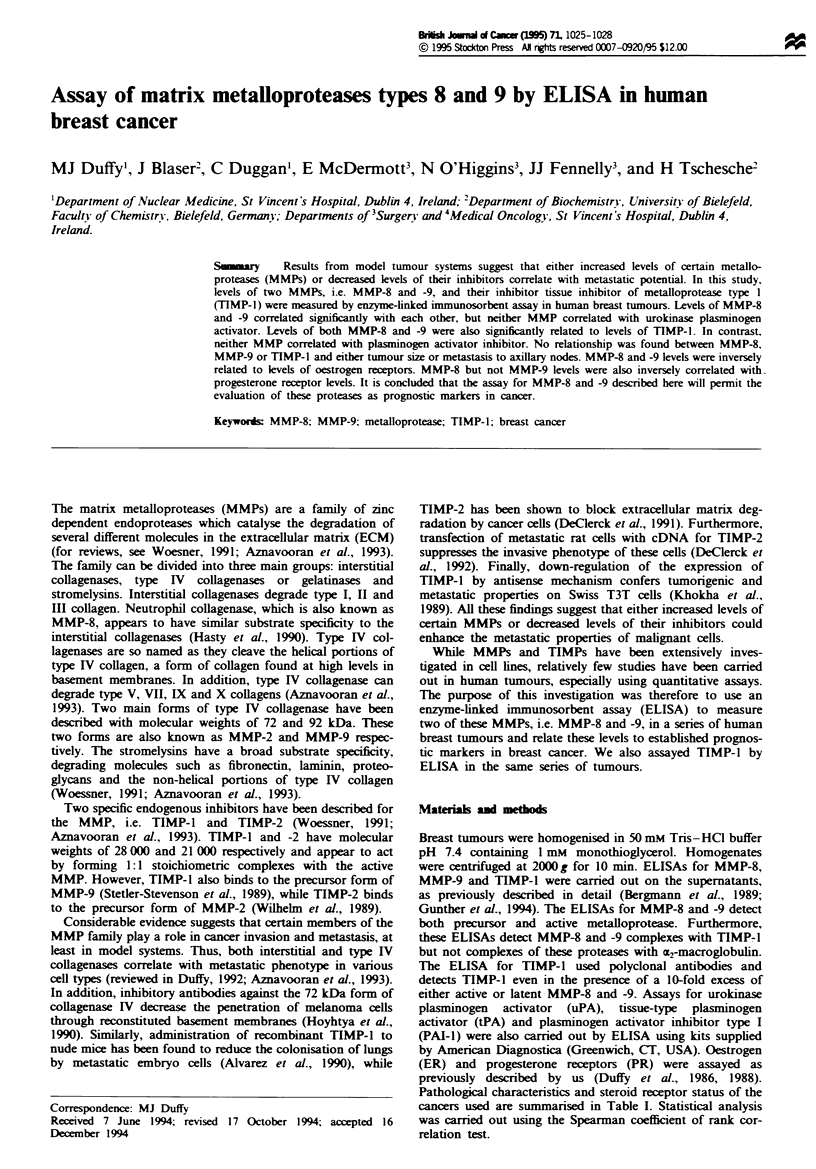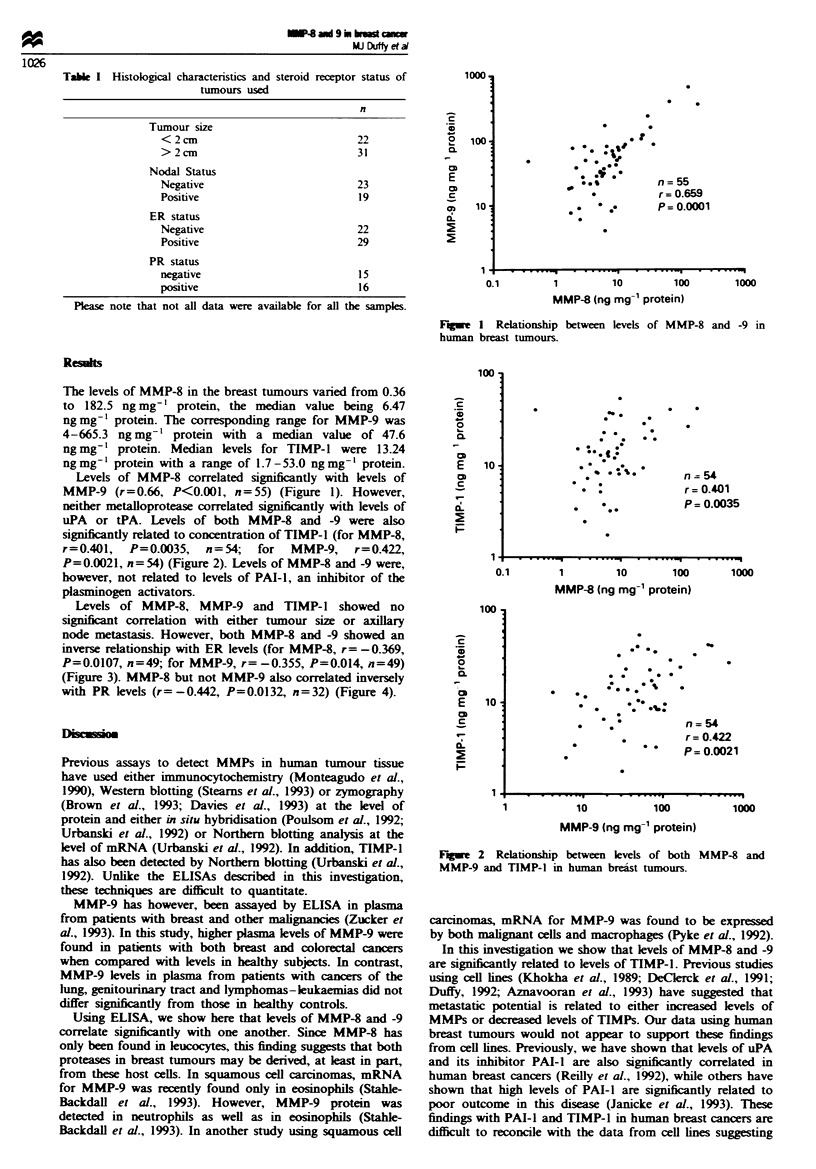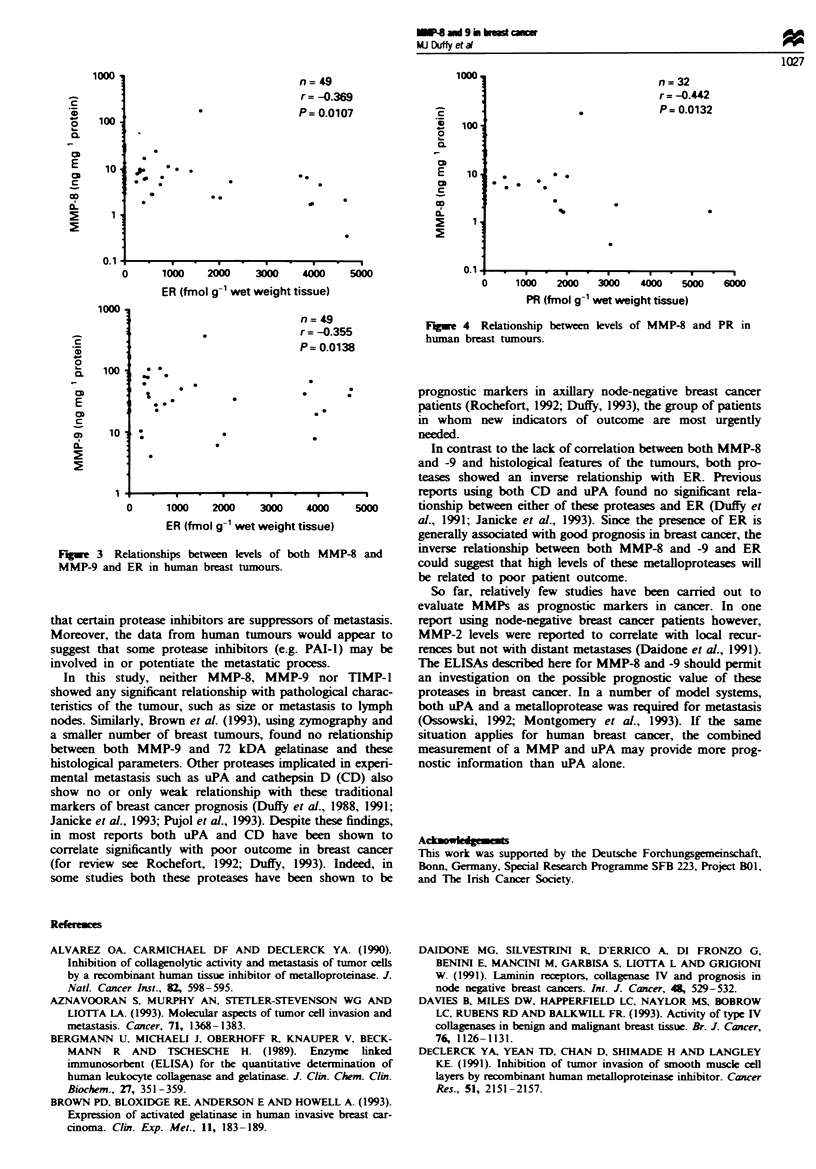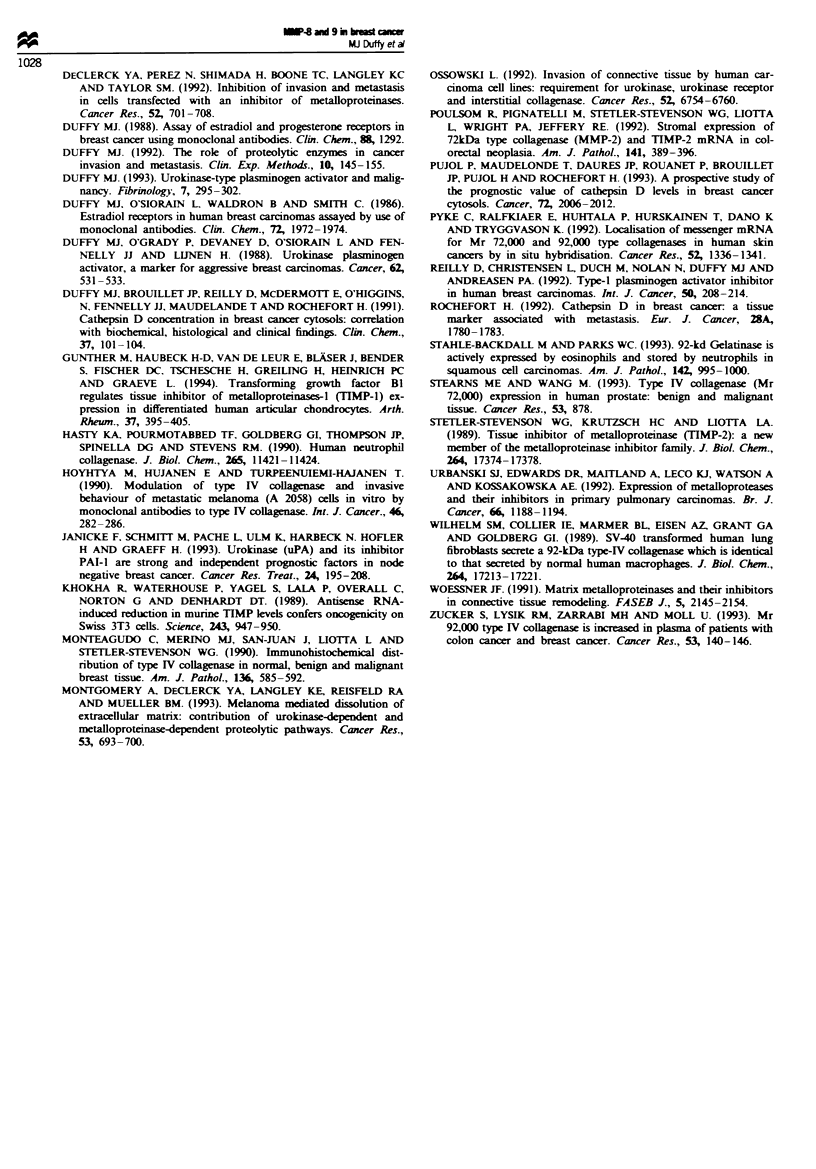# Assay of matrix metalloproteases types 8 and 9 by ELISA in human breast cancer.

**DOI:** 10.1038/bjc.1995.197

**Published:** 1995-05

**Authors:** M. J. Duffy, J. Blaser, C. Duggan, E. McDermott, N. O'Higgins, J. J. Fennelly, H. Tschesche

**Affiliations:** Department of Nuclear Medicine, St Vincent's Hospital, Dublin, Ireland.

## Abstract

Results from model tumour systems suggest that either increased levels of certain metalloproteases (MMPs) or decreased levels of their inhibitors correlate with metastatic potential. In this study, levels of two MMPs, i.e. MMP-8 and -9, and their inhibitor tissue inhibitor of metalloprotease type 1 (TIMP-1) were measured by enzyme-linked immunosorbent assay in human breast tumours. Levels of MMP-8 and -9 correlated significantly with each other, but neither MMP correlated with urokinase plasminogen activator. Levels of both MMP-8 and -9 were also significantly related to levels of TIMP-1. In contrast, neither MMP correlated with plasminogen activator inhibitor. No relationship was found between MMP-8, MMP-9 or TIMP-1 and either tumour size or metastasis to axillary nodes. MMP-8 and -9 levels were inversely related to levels of oestrogen receptors. MMP-8 but not MMP-9 levels were also inversely correlated with progesterone receptor levels. It is concluded that the assay for MMP-8 and -9 described here will permit the evaluation of these proteases as prognostic markers in cancer.


© 1995 Stockton Press All rights reserved 0007-0920/95 $12.00

### Assay of matrix metalloproteases types 8 and 9 by ELISA in human breast cancer

MJ Duffy[1], J Blaser[2], C Duggan[1], E McDermott[3], N O'Higgins[3], JJ Fennelly[3], and H Tschesche[2]

[1]*Department of Nuclear Medicine, St Vincent's Hospital, Dublin 4, Ireland;* [2]*Department of Biochemistry, University of Bielefeld, Faculty of Chemistry, Bielefeld, Germany; Departments of* [3]*Surgery and* [4]*Medical Oncology, St Vincent's Hospital, Dublin 4, Ireland.*

**Summary** Results from model tumour systems suggest that either increased levels of certain metalloproteases (MMPs) or decreased levels of their inhibitors correlate with metastatic potential. In this study, levels of two MMPs, i.e. MMP-8 and -9, and their inhibitor tissue inhibitor of metalloprotease type 1 (TIMP-1) were measured by enzyme-linked immunosorbent assay in human breast tumours. Levels of MMP-8 and -9 correlated significantly with each other, but neither MMP correlated with urokinase plasminogen activator. Levels of both MMP-8 and -9 were also significantly related to levels of TIMP-1. In contrast, neither MMP correlated with plasminogen activator inhibitor. No relationship was found between MMP-8, MMP-9 or TIMP-1 and either tumour size or metastasis to axillary nodes. MMP-8 and -9 levels were inversely related to levels of oestrogen receptors. MMP-8 but not MMP-9 levels were also inversely correlated with progesterone receptor levels. It is concluded that the assay for MMP-8 and -9 described here will permit the evaluation of these proteases as prognostic markers in cancer.

**Keywords:** MMP-8; MMP-9; metalloprotease; TIMP-1; breast cancer

The matrix metalloproteases (MMPs) are a family of zinc dependent endoproteases which catalyse the degradation of several different molecules in the extracellular matrix (ECM) (for reviews, see Woesner, 1991; Aznavooran *et al.*, 1993). The family can be divided into three main groups: interstitial collagenases, type IV collagenases or gelatinases and stromelysins. Interstitial collagenases degrade type I, II and III collagen. Neutrophil collagenase, which is also known as MMP-8, appears to have similar substrate specificity to the interstitial collagenases (Hasty *et al.*, 1990). Type IV collagenases are so named as they cleave the helical portions of type IV collagen, a form of collagen found at high levels in basement membranes. In addition, type IV collagenase can degrade type V, VII, IX and X collagens (Aznavooran *et al.*, 1993). Two main forms of type IV collagenase have been described with molecular weights of 72 and 92 kDa. These two forms are also known as MMP-2 and MMP-9 respectively. The stromelysins have a broad substrate specificity, degrading molecules such as fibronectin, laminin, proteoglycans and the non-helical portions of type IV collagen (Woessner, 1991; Aznavooran *et al.*, 1993).

Two specific endogenous inhibitors have been described for the MMP, i.e. TIMP-1 and TIMP-2 (Woessner, 1991; Aznavooran *et al.*, 1993). TIMP-1 and -2 have molecular weights of 28 000 and 21 000 respectively and appear to act by forming 1:1 stoichiometric complexes with the active MMP. However, TIMP-1 also binds to the precursor form of MMP-9 (Stetler-Stevenson *et al.*, 1989), while TIMP-2 binds to the precursor form of MMP-2 (Wilhelm *et al.*, 1989).

Considerable evidence suggests that certain members of the MMP family play a role in cancer invasion and metastasis, at least in model systems. Thus, both interstitial and type IV collagenases correlate with metastatic phenotype in various cell types (reviewed in Duffy, 1992; Aznavooran *et al.*, 1993). In addition, inhibitory antibodies against the 72 kDa form of collagenase IV decrease the penetration of melanoma cells through reconstituted basement membranes (Hoyhtya *et al.*, 1990). Similarly, administration of recombinant TIMP-1 to nude mice has been found to reduce the colonisation of lungs by metastatic embryo cells (Alvarez *et al.*, 1990), while TIMP-2 has been shown to block extracellular matrix degradation by cancer cells (DeClerck *et al.*, 1991). Furthermore, transfection of metastatic rat cells with cDNA for TIMP-2 suppresses the invasive phenotype of these cells (DeClerck *et al.*, 1992). Finally, down-regulation of the expression of TIMP-1 by antisense mechanism confers tumorigenic and metastatic properties on Swiss T3T cells (Khokha *et al.*, 1989). All these findings suggest that either increased levels of certain MMPs or decreased levels of their inhibitors could enhance the metastatic properties of malignant cells.

While MMPs and TIMPs have been extensively investigated in cell lines, relatively few studies have been carried out in human tumours, especially using quantitative assays. The purpose of this investigation was therefore to use an enzyme-linked immunosorbent assay (ELISA) to measure two of these MMPs, i.e. MMP-8 and -9, in a series of human breast tumours and relate these levels to established prognostic markers in breast cancer. We also assayed TIMP-1 by ELISA in the same series of tumours.

### Materials and methods

Breast tumours were homogenised in 50 mM Tris-HCl buffer pH 7.4 containing 1 mM monothioglycerol. Homogenates were centrifuged at 2000 *g* for 10 min. ELISAs for MMP-8, MMP-9 and TIMP-1 were carried out on the supernatants, as previously described in detail (Bergmann *et al.*, 1989; Gunther *et al.*, 1994). The ELISAs for MMP-8 and -9 detect both precursor and active metalloprotease. Furthermore, these ELISAs detect MMP-8 and -9 complexes with TIMP-1 but not complexes of these proteases with $\alpha_2$-macroglobulin. The ELISA for TIMP-1 used polyclonal antibodies and detects TIMP-1 even in the presence of a 10-fold excess of either active or latent MMP-8 and -9. Assays for urokinase plasminogen activator (uPA), tissue-type plasminogen activator (tPA) and plasminogen activator inhibitor type I (PAI-1) were also carried out by ELISA using kits supplied by American Diagnostica (Greenwich, CT, USA). Oestrogen (ER) and progesterone receptors (PR) were assayed as previously described by us (Duffy *et al.*, 1986, 1988). Pathological characteristics and steroid receptor status of the cancers used are summarised in Table I. Statistical analysis was carried out using the Spearman coefficient of rank correlation test.

Correspondence: MJ Duffy
Received 7 June 1994; revised 17 October 1994; accepted 16 December 1994

**Table I** Histological characteristics and steroid receptor status of tumours used

| | *n* |
|---|---|
| Tumour size | |
| < 2 cm | 22 |
| > 2 cm | 31 |
| Nodal Status | |
| Negative | 23 |
| Positive | 19 |
| ER status | |
| Negative | 22 |
| Positive | 29 |
| PR status | |
| negative | 15 |
| positive | 16 |

Please note that not all data were available for all the samples.

### Results

The levels of MMP-8 in the breast tumours varied from 0.36 to 182.5 ng mg$^{-1}$ protein, the median value being 6.47 ng mg$^{-1}$ protein. The corresponding range for MMP-9 was 4–665.3 ng mg$^{-1}$ protein with a median value of 47.6 ng mg$^{-1}$ protein. Median levels for TIMP-1 were 13.24 ng mg$^{-1}$ protein with a range of 1.7–53.0 ng mg$^{-1}$ protein.

Levels of MMP-8 correlated significantly with levels of MMP-9 ($r$=0.66, $P$<0.001, $n$=55) (Figure 1). However, neither metalloprotease correlated significantly with levels of uPA or tPA. Levels of both MMP-8 and -9 were also significantly related to concentration of TIMP-1 (for MMP-8, $r$=0.401, $P$=0.0035, $n$=54; for MMP-9, $r$=0.422, $P$=0.0021, $n$=54) (Figure 2). Levels of MMP-8 and -9 were, however, not related to levels of PAI-1, an inhibitor of the plasminogen activators.

Levels of MMP-8, MMP-9 and TIMP-1 showed no significant correlation with either tumour size or axillary node metastasis. However, both MMP-8 and -9 showed an inverse relationship with ER levels (for MMP-8, $r$=−0.369, $P$=0.0107, $n$=49; for MMP-9, $r$=−0.355, $P$=0.014, $n$=49) (Figure 3). MMP-8 but not MMP-9 also correlated inversely with PR levels ($r$=−0.442, $P$=0.0132, $n$=32) (Figure 4).

### Discussion

Previous assays to detect MMPs in human tumour tissue have used either immunocytochemistry (Monteagudo *et al.*, 1990), Western blotting (Stearns *et al.*, 1993) or zymography (Brown *et al.*, 1993; Davies *et al.*, 1993) at the level of protein and either *in situ* hybridisation (Poulsom *et al.*, 1992; Urbanski *et al.*, 1992) or Northern blotting analysis at the level of mRNA (Urbanski *et al.*, 1992). In addition, TIMP-1 has also been detected by Northern blotting (Urbanski *et al.*, 1992). Unlike the ELISAs described in this investigation, these techniques are difficult to quantitate.

MMP-9 has however, been assayed by ELISA in plasma from patients with breast and other malignancies (Zucker *et al.*, 1993). In this study, higher plasma levels of MMP-9 were found in patients with both breast and colorectal cancers when compared with levels in healthy subjects. In contrast, MMP-9 levels in plasma from patients with cancers of the lung, genitourinary tract and lymphomas–leukaemias did not differ significantly from those in healthy controls.

Using ELISA, we show here that levels of MMP-8 and -9 correlate significantly with one another. Since MMP-8 has only been found in leucocytes, this finding suggests that both proteases in breast tumours may be derived, at least in part, from these host cells. In squamous cell carcinomas, mRNA for MMP-9 was recently found only in eosinophils (Stahle-Backdall *et al.*, 1993). However, MMP-9 protein was detected in neutrophils as well as in eosinophils (Stahle-Backdall *et al.*, 1993). In another study using squamous cell

**Figure 1** Relationship between levels of MMP-8 and -9 in human breast tumours.

**Figure 2** Relationship between levels of both MMP-8 and MMP-9 and TIMP-1 in human breast tumours.

carcinomas, mRNA for MMP-9 was found to be expressed by both malignant cells and macrophages (Pyke *et al.*, 1992).

In this investigation we show that levels of MMP-8 and -9 are significantly related to levels of TIMP-1. Previous studies using cell lines (Khokha *et al.*, 1989; DeClerck *et al.*, 1991; Duffy, 1992; Aznavooran *et al.*, 1993) have suggested that metastatic potential is related to either increased levels of MMPs or decreased levels of TIMPs. Our data using human breast tumours would not appear to support these findings from cell lines. Previously, we have shown that levels of uPA and its inhibitor PAI-1 are also significantly correlated in human breast cancers (Reilly *et al.*, 1992), while others have shown that high levels of PAI-1 are significantly related to poor outcome in this disease (Janicke *et al.*, 1993). These findings with PAI-1 and TIMP-1 in human breast cancers are difficult to reconcile with the data from cell lines suggesting

**Figure 3** Relationships between levels of both MMP-8 and MMP-9 and ER in human breast tumours.

**Figure 4** Relationship between levels of MMP-8 and PR in human breast tumours.

that certain protease inhibitors are suppressors of metastasis. Moreover, the data from human tumours would appear to suggest that some protease inhibitors (e.g. PAI-1) may be involved in or potentiate the metastatic process.

In this study, neither MMP-8, MMP-9 nor TIMP-1 showed any significant relationship with pathological characteristics of the tumour, such as size or metastasis to lymph nodes. Similarly, Brown *et al.* (1993), using zymography and a smaller number of breast tumours, found no relationship between both MMP-9 and 72 kDA gelatinase and these histological parameters. Other proteases implicated in experimental metastasis such as uPA and cathepsin D (CD) also show no or only weak relationship with these traditional markers of breast cancer prognosis (Duffy *et al.*, 1988, 1991; Janicke *et al.*, 1993; Pujol *et al.*, 1993). Despite these findings, in most reports both uPA and CD have been shown to correlate significantly with poor outcome in breast cancer (for review see Rochefort, 1992; Duffy, 1993). Indeed, in some studies both these proteases have been shown to be prognostic markers in axillary node-negative breast cancer patients (Rochefort, 1992; Duffy, 1993), the group of patients in whom new indicators of outcome are most urgently needed.

In contrast to the lack of correlation between both MMP-8 and -9 and histological features of the tumours, both proteases showed an inverse relationship with ER. Previous reports using both CD and uPA found no significant relationship between either of these proteases and ER (Duffy *et al.*, 1991; Janicke *et al.*, 1993). Since the presence of ER is generally associated with good prognosis in breast cancer, the inverse relationship between both MMP-8 and -9 and ER could suggest that high levels of these metalloproteases will be related to poor patient outcome.

So far, relatively few studies have been carried out to evaluate MMPs as prognostic markers in cancer. In one report using node-negative breast cancer patients however, MMP-2 levels were reported to correlate with local recurrences but not with distant metastases (Daidone *et al.*, 1991). The ELISAs described here for MMP-8 and -9 should permit an investigation on the possible prognostic value of these proteases in breast cancer. In a number of model systems, both uPA and a metalloprotease was required for metastasis (Ossowski, 1992; Montgomery *et al.*, 1993). If the same situation applies for human breast cancer, the combined measurement of a MMP and uPA may provide more prognostic information than uPA alone.

**Acknowledgements**

This work was supported by the Deutsche Forchungsgemeinschaft, Bonn, Germany, Special Research Programme SFB 223, Project B01, and The Irish Cancer Society.

**References**

ALVAREZ OA, CARMICHAEL DF AND DECLERCK YA. (1990). Inhibition of collagenolytic activity and metastasis of tumor cells by a recombinant human tissue inhibitor of metalloproteinase. *J. Natl. Cancer Inst.*, **82**, 598-595.

AZNAVOORAN S, MURPHY AN, STETLER-STEVENSON WG AND LIOTTA LA. (1993). Molecular aspects of tumor cell invasion and metastasis. *Cancer*, **71**, 1368-1383.

BERGMANN U, MICHAELI J, OBERHOFF R, KNAUPER V, BECKMANN R AND TSCHESCHE H. (1989). Enzyme linked immunosorbent (ELISA) for the quantitative determination of human leukocyte collagenase and gelatinase. *J. Clin. Chem. Clin. Biochem.*, **27**, 351-359.

BROWN PD, BLOXIDGE RE, ANDERSON E AND HOWELL A. (1993). Expression of activated gelatinase in human invasive breast carcinoma. *Clin. Exp. Met.*, **11**, 183-189.

DAIDONE MG, SILVESTRINI R, D'ERRICO A, DI FRONZO G, BENINI E, MANCINI M, GARBISA S, LIOTTA L AND GRIGIONI W. (1991). Laminin receptors, collagenase IV and prognosis in node negative breast cancers. *Int. J. Cancer*, **48**, 529-532.

DAVIES B, MILES DW, HAPPERFIELD LC, NAYLOR MS, BOBROW LC, RUBENS RD AND BALKWILL FR. (1993). Activity of type IV collagenases in benign and malignant breast tissue. *Br. J. Cancer*, **76**, 1126-1131.

DECLERCK YA, YEAN TD, CHAN D, SHIMADE H AND LANGLEY KE. (1991). Inhibition of tumor invasion of smooth muscle cell layers by recombinant human metalloproteinase inhibitor. *Cancer Res.*, **51**, 2151-2157.

DECLERCK YA, PEREZ N, SHIMADA H, BOONE TC, LANGLEY KC AND TAYLOR SM. (1992). Inhibition of invasion and metastasis in cells transfected with an inhibitor of metalloproteinases. *Cancer Res.*, **52**, 701–708.

DUFFY MJ. (1988). Assay of estradiol and progesterone receptors in breast cancer using monoclonal antibodies. *Clin. Chem.*, **88**, 1292.

DUFFY MJ. (1992). The role of proteolytic enzymes in cancer invasion and metastasis. *Clin. Exp. Methods.*, **10**, 145–155.

DUFFY MJ. (1993). Urokinase-type plasminogen activator and malignancy. *Fibrinology*, **7**, 295–302.

DUFFY MJ, O'SIORAIN L, WALDRON B AND SMITH C. (1986). Estradiol receptors in human breast carcinomas assayed by use of monoclonal antibodies. *Clin. Chem.*, **72**, 1972–1974.

DUFFY MJ, O'GRADY P, DEVANEY D, O'SIORAIN L AND FENNELLY JJ AND LIJNEN H. (1988). Urokinase plasminogen activator, a marker for aggressive breast carcinomas. *Cancer*, **62**, 531–533.

DUFFY MJ, BROUILLET JP, REILLY D, MCDERMOTT E, O'HIGGINS, N, FENNELLY JJ, MAUDELANDE T AND ROCHEFORT H. (1991). Cathepsin D concentration in breast cancer cytosols: correlation with biochemical, histological and clinical findings. *Clin. Chem.*, **37**, 101–104.

GUNTHER M, HAUBECK H-D, VAN DE LEUR E, BLÄSER J, BENDER S, FISCHER DC, TSCHESCHE H, GREILING H, HEINRICH PC AND GRAEVE L. (1994). Transforming growth factor B1 regulates tissue inhibitor of metalloproteinases-1 (TIMP-1) expression in differentiated human articular chondrocytes. *Arth. Rheum.*, **37**, 395–405.

HASTY KA, POURMOTABBED TF, GOLDBERG GI, THOMPSON JP, SPINELLA DG AND STEVENS RM. (1990). Human neutrophil collagenase. *J. Biol. Chem.*, **265**, 11421–11424.

HOYHTYA M, HUJANEN E AND TURPEENUIEMI-HAJANEN T. (1990). Modulation of type IV collagenase and invasive behaviour of metastatic melanoma (A 2058) cells in vitro by monoclonal antibodies to type IV collagenase. *Int. J. Cancer.*, **46**, 282–286.

JANICKE F, SCHMITT M, PACHE L, ULM K, HARBECK N, HOFLER H AND GRAEFF H. (1993). Urokinase (uPA) and its inhibitor PAI-1 are strong and independent prognostic factors in node negative breast cancer. *Cancer Res. Treat.*, **24**, 195–208.

KHOKHA R, WATERHOUSE P, YAGEL S, LALA P, OVERALL C, NORTON G AND DENHARDT DT. (1989). Antisense RNA-induced reduction in murine TIMP levels confers oncogenicity on Swiss 3T3 cells. *Science*, **243**, 947–950.

MONTEAGUDO C, MERINO MJ, SAN-JUAN J, LIOTTA L AND STETLER-STEVENSON WG. (1990). Immunohistochemical distribution of type IV collagenase in normal, benign and malignant breast tissue. *Am. J. Pathol.*, **136**, 585–592.

MONTGOMERY A, DECLERCK YA, LANGLEY KE, REISFELD RA AND MUELLER BM. (1993). Melanoma mediated dissolution of extracellular matrix: contribution of urokinase-dependent and metalloproteinase-dependent proteolytic pathways. *Cancer Res.*, **53**, 693–700.

OSSOWSKI L. (1992). Invasion of connective tissue by human carcinoma cell lines: requirement for urokinase, urokinase receptor and interstitial collagenase. *Cancer Res.*, **52**, 6754–6760.

POULSOM R, PIGNATELLI M, STETLER-STEVENSON WG, LIOTTA L, WRIGHT PA, JEFFERY RE. (1992). Stromal expression of 72kDa type collagenase (MMP-2) and TIMP-2 mRNA in colorectal neoplasia. *Am. J. Pathol.*, **141**, 389–396.

PUJOL P, MAUDELONDE T, DAURES JP, ROUANET P, BROUILLET JP, PUJOL H AND ROCHEFORT H. (1993). A prospective study of the prognostic value of cathepsin D levels in breast cancer cytosols. *Cancer*, **72**, 2006–2012.

PYKE C, RALFKIAER E, HUHTALA P, HURSKAINEN T, DANO K AND TRYGGVASON K. (1992). Localisation of messenger mRNA for Mr 72,000 and 92,000 type collagenases in human skin cancers by in situ hybridisation. *Cancer Res.*, **52**, 1336–1341.

REILLY D, CHRISTENSEN L, DUCH M, NOLAN N, DUFFY MJ AND ANDREASEN PA. (1992). Type-1 plasminogen activator inhibitor in human breast carcinomas. *Int. J. Cancer*, **50**, 208–214.

ROCHEFORT H. (1992). Cathepsin D in breast cancer: a tissue marker associated with metastasis. *Eur. J. Cancer*, **28A**, 1780–1783.

STAHLE-BACKDALL M AND PARKS WC. (1993). 92-kd Gelatinase is actively expressed by eosinophils and stored by neutrophils in squamous cell carcinomas. *Am. J. Pathol.*, **142**, 995–1000.

STEARNS ME AND WANG M. (1993). Type IV collagenase (Mr 72,000) expression in human prostate: benign and malignant tissue. *Cancer Res.*, **53**, 878.

STETLER-STEVENSON WG, KRUTZSCH HC AND LIOTTA LA. (1989). Tissue inhibitor of metalloproteinase (TIMP-2): a new member of the metalloproteinase inhibitor family. *J. Biol. Chem.*, **264**, 17374–17378.

URBANSKI SJ, EDWARDS DR, MAITLAND A, LECO KJ, WATSON A AND KOSSAKOWSKA AE. (1992). Expression of metalloproteases and their inhibitors in primary pulmonary carcinomas. *Br. J. Cancer*, **66**, 1188–1194.

WILHELM SM, COLLIER IE, MARMER BL, EISEN AZ, GRANT GA AND GOLDBERG GI. (1989). SV-40 transformed human lung fibroblasts secrete a 92-kDa type-IV collagenase which is identical to that secreted by normal human macrophages. *J. Biol. Chem.*, **264**, 17213–17221.

WOESSNER JF. (1991). Matrix metalloproteinases and their inhibitors in connective tissue remodeling. *FASEB J.*, **5**, 2145–2154.

ZUCKER S, LYSIK RM, ZARRABI MH AND MOLL U. (1993). Mr 92,000 type IV collagenase is increased in plasma of patients with colon cancer and breast cancer. *Cancer Res.*, **53**, 140–146.